# *Aspergillus aculeatus* enhances nutrient uptake and forage quality in bermudagrass by increasing phosphorus and potassium availability

**DOI:** 10.3389/fpls.2023.1165567

**Published:** 2023-04-25

**Authors:** Xiaoning Li, Ting Zhang, Ying Xue, Xiao Xu, Xinyu Cui, Jinmin Fu

**Affiliations:** Coastal Salinity Tolerant Grass Engineering and Technology Research Center, Ludong University, Yantai, China

**Keywords:** *Aspergillus aculeatus*, bermudagrass, potassium, phosphorus, P or K deficiency stress

## Abstract

**Introduction:**

Potassium and phosphorus are essential macronutrients for plant growth and development. However, most P and K exist in insoluble forms, which are difficult for plants to directly absorb and utilize, thereby resulting in growth retardation of plants under P or K deficiency stress. The *Aspergillus aculeatus* fungus has growth-promoting characteristics and the ability to dissolve P and K.

**Methods:**

Here, to investigate the physiological effects of *A. aculeatus* on bermudagrass under P or K deficiency, *A. aculeatus* and bermudagrass were used as experimental materials.

**Results and discussion:**

The results showed that *A. aculeatus* could promote tolerance to P or K deficiency stress in bermudagrass, decrease the rate of leaf death, and increase the contents of crude fat as well as crude protein. In addition, *A. aculeatus* significantly enhanced the chlorophyll a+b and carotenoid contents. Moreover, under P or K deficiency stress, bermudagrass inoculated with *A. aculeatus* showed higher N, P, and K contents than non-inoculated plants. Furthermore, exogenous *A. aculeatus* markedly decreased the H_2_O_2_ level and CAT and POD activities. Based on our results, *A. aculeatus* could effectively improve the forage quality of bermudagrass and alleviate the negative effects of P or K deficiency stress, thereby playing a positive economic role in the forage industry.

## 1 Introduction

Phosphorus (P) and potassium (K) are the major macronutrients for plant growth and development (Wang et al., 2021). P and K play dominant roles in maintaining a variety of physiological metabolic processes in plants, including nucleic acid synthesis, energy metabolites, membrane lipids, photosynthesis, membrane polarization, and protein biosynthesis (Clarkson and Hanson, 1980; Poirier and Bucher, 2002). However, P and K in soil exist in the form of insoluble K, which cannot be directly absorbed and used by plants (Illmer and Schinner, 1995; Meena et al., 2016). Therefore, it is of great interest to investigate management strategies that can improve the availability of phosphorus and potassium.

As fundamental mineral nutrients, P and K have been documented to be involved in plant photosynthesis processes (Wang et al., 2012; Krishnaraj and Dahale, 2014). Previous investigators proposed that P deficiency triggered reddish leaf and necrosis on the tips of old leaves and could result in a decrease in the maximum PSII efficiency and electron transport rate (Guo et al., 2002; Luiz et al., 2018). Chlorosis along the leaf margins is an obvious symptom of K deficiency. In severe cases, the leaf will turn yellow and fall off (Meena et al., 2016). Previous research has reported that K deficiency disrupts leaf photosynthetic performance and causes a lower net photosynthetic rate and stomatal conductance, which directly affects the yield of plants (Zhao et al., 2016).

A deficiency of mineral nutrition in plants could cause oxidative stress due to the disequilibrium between the scavenging and production of excess reactive oxygen species (ROS) (Cakmak, 2005; Shin et al., 2005). An excess of ROS triggers protein denaturation, cell membrane lipid peroxidation, and nucleic acid degradation, which restrains normal cellular physiological processes (Mittova et al., 2002). Fortunately, ROS can be scavenged through synergistic and interactive enzymatic and non-enzymatic antioxidant defense systems. The enzymatic systems mainly include peroxidase (POD), superoxide dismutase (SOD), catalase (CAT), ascorbate peroxidase (APX), and glutathione peroxidase (GPX) (Apel and Hirt, 2004).

Whenever the soil cannot adequately supply the P and K required for plant growth, people must supplement soil reserves with chemical P and K fertilizers. However, not all fertilizers that have been applied to the soil are fully taken up and utilized by plants. A portion of the applied fertilizers are left behind, thereby causing environmental contamination, such as eutrophication, and soil fertility depletion (Sharma et al., 2013; Meena et al., 2016; Kumar et al., 2021). Therefore, the emergence of phosphorus and potassium fertilizers has not given the ultimate solution. Such environmental concerns have triggered the exploration of sustainable P and K nutrition in plants. Given this circumstance, phosphate-solubilizing microorganisms have been considered an eco-friendly and cost-effective approach for the supply of P nutrition to plants (Sharma et al., 2013; Meena et al., 2016; Garcia et al., 2017). Mounting evidence suggests that P-solubilizing microorganisms (such as *Azotobacter*, *Bradyrhizobium*, *Penicillium*, and *Aspergillus*) can convert insoluble P into the bioavailable form through various mechanisms of solubilization and mineralization (Bargaz et al., 2018). Similarly, diverse groups of K-solubilizing microorganisms (*Bacillus mucilaginosus*, *Aspergillus terreus*, and *Aspergillus niger*) were proven to be involved in solubilizing the insoluble forms of K into available forms that are directly absorbed and utilized by plants (Prajapati and Modi, 2012; Gundala et al., 2013; Zarjani et al., 2013). Therefore, improvement of P and K utilization efficiency provides a potential strategy to overcome the adverse effects of P and K deficiencies. Investigation of the characteristics of fungi in dissolving P and K is an important prerequisite to improve plant nutrient utilization efficiency.

*Aspergillus aculeatus* is isolated from the rhizosphere of bermudagrass in heavy metal-contaminated areas (Xie et al., 2014). Our previous study proved that *A. aculeatus* can facilitate plant growth by producing indole-3-acetic acid, ACC deaminase, and siderophores (Xie et al., 2017). In addition, we have demonstrated that the fungus possessed P- and K-solubilizing characteristics, which accelerated the uptake and utilization of P and K nutrient elements, thereby promoting the growth and development of plants (Li et al., 2019; Li et al., 2021). However, the critical function of *A. aculeatus* in increasing the performance of bermudagrass exposed to P and K deficiency is still ambiguous. Bermudagrass [*Cynodon dactylon* (L.) Pers] is a typical warm season turfgrass and forage and is widely used in urban greening, sports fields, slope protection, and animal husbandry due to its high reproduction rate, short-term turf establishment, and strong mechanical stress resistance (Fan et al., 2014; Shi et al., 2014).

The objective of this experiment was to explore *A. aculeatus*-mediated protective responses to P or K deficiency stress in bermudagrass. We measured important physiological indicators of P or K deficiency stress, such as biomass, dead leaf rate, chlorophyll, carotenoids, forage quality, ion content, and antioxidant enzyme activities.

## 2 Materials and methods

### 2.1 Culture of bermudagrass and *Aspergillus aculeatus*

The experimental materials were obtained from the artificially bred bermudagrass ‘Wrangler’ in the coastal grass germplasm resources and breeding base located in Ludong University, Yantai City, Shandong Province. The fourth to sixth stem segments (three stem segments) of the biological upper end of the bermudagrass were selected, and stems with the same length and thickness were inserted into the pots (10 cm in diameter and 15 cm in height) on 25 July 2021. All the materials were placed in a plant growth incubator at 30°C/25°C (day/night), with a 14-h photoperiod, 400 μmol photons m^−2^ s^−1^ of light intensity, and 60% relative humidity for 7 weeks to establish the roots and leaves. The grass was irrigated with 0.5× Hoagland nutrient solution every 2 days and cut based on a one-third principle. *Aspergillus aculeatus* was cultured and massively propagated in a Martin liquid medium according to our previous study (Xie et al., 2017).

### 2.2 Experimental treatment

A mixture of sand and sawdust (v:v = 3:1) was used as the growth substance for this experiment. Subsequently, on 10 September 2021, all the substances were sterilized at 127°C for 1 h in an autoclave and then dispensed into 24 pots (10 cm in diameter and 18 cm in height) with 550 g of the mixed matrix. On 12 September 2021, all the roots and shoots of the materials were washed and trimmed, and the length or height was maintained at 8 and 10 cm, respectively. The initial weight was recorded as *W*_0_, the trimmed plants were transferred into the above pots, and the 0.5× Hoagland nutrient solution (not present at the source of phosphorus or potassium, i.e., P or K deficiency treatment), was used to irrigate the plants every 2 days (100 ml pot^−1^). For the P deficiency experiment, tricalcium phosphate was evenly mixed into the growth substance at a concentration of 5 g per pot before the plants were transplanted. Four groups and three replicates were designed, including P_0_A_0_ (no tricalcium phosphate and *A. aculeatus* treatment), P_0_A_1_ (only *A. aculeatus*), P_1_A_0_ (only tricalcium phosphate), and P_1_A_1_ (tricalcium phosphate and *A. aculeatus* treatment). In the same way, for the K deficiency experiment, K-feldspar was evenly mixed into the growth substance at a concentration of 5 g per pot before the plants were transplanted. The four groups and three replicates were designed, including K_0_A_0_ (no K-feldspar and *A. aculeatus* treatment), K_0_A_1_ (only *A. aculeatus*), K_1_A_0_ (only K-feldspar), and K_1_A_1_ (K-feldspar and *A. aculeatus* treatment). For the inoculated *A. aculeatus* treatment groups, 100 ml of fungal spore suspension was inoculated into the growth substances. The treatment lasted for 4 weeks, and then the plants were harvested for further analysis on 10 October 2021.

### 2.3 Determination of growth parameters and forage quality

For growth biomass (GB) determination, the whole bermudagrass (including aboveground and underground) were washed and weighed (*W*_1_) at the end of the experiment. GB (kg day^−1^) = (*W*_1_ – *W*_0_)/*D*. *W*_0_ is the fresh weight before treatment, *W*_1_ is the fresh weight at the end of treatment, and *D* is the treatment days. To calculate the dead leaf rate (DLR), the leaf biomass (LB) and dead leaf biomass (DLB) of bermudagrass were weighed and recorded. DLR = DLB/LB × 100%.

For crude protein content measurement, the dried samples (0.20 g) were digested with 10 ml of H_2_SO_4_ using a graphite digestion apparatus (SH220N; Jinan Hanon, Shandong, China). The crude protein content was measured by an automatic Kjeldahl apparatus (Hanon K9860). Crude protein content (%) = N content × 6.25 × 100%.

To assess the crude fat content, the dried sample powder was weighed and measured with the Soxhlet extraction method. The samples were mixed with 50 ml of petroleum ether and dried in an oven at 120°C for 3 h with a Soxhlet apparatus (SOX406; Jinan Hanon, Shandong, China), and the residue was weighed and recorded.

### 2.4 Determination of ion content

To measure the N, P, and K contents, dried samples of the leaves (0.20 g) were digested with 10 ml of 99% sulfuric acid (H_2_SO_4_) in a graphite digestion apparatus. The contents of P and N were determined by a fully automatic intermittent chemical analyzer (SmartChem 200; AMS Alliance, Guidonia, Rome, Italy) (Zhang et al., 2020). The K content was assessed with flame photometry (Jackson, 1973).

### 2.5 Determination of chlorophyll and carotenoid content

Chlorophyll and carotenoids were extracted with dimethyl sulfoxide from all leaf segments (200 mg) and were determined using ultraviolet spectrophotometry (UV1700; Meixi, Shanghai, China) according to the method of Wellburn (1994).

### 2.6 Determination of antioxidant enzyme activity

Antioxidant enzymes were extracted at 4°C using 200 mg of tissue from the fresh samples of bermudagrass leaves. Plant samples were homogenized with 8 ml of phosphate buffer (pH = 7.8) and were centrifuged at 12,000 rpm for 20 min, and then the supernatants were collected for the determination of the activities of antioxidant enzymes. POD activity was assayed by measuring the increase in absorbance at 470 nm with guaiacol as the substrate. CAT activity was determined by calculating the substrate consumption of H_2_O_2_ in absorbance at 240 nm (Hu et al., 2011). The content of H_2_O_2_ was determined according to the method of the hydrogen peroxide kit (Nanjing Jiancheng Bioengineering Institute, A064, Nanjing, China), and the absorbance value at 405 nm was measured with a spectrophotometer to calculate the content of H_2_O_2_.

### 2.7 Statistical analysis

The raw data of the whole experiment were statistically analyzed with one-way ANOVA using SPSS software (Statistical Product and Service Solutions, version 20; IBM, Chicago, United States). The overall significance of the treatment was tested by the SNK test at the *p <*0.05 level. Correlation analysis was performed using the Pearson method.

## 3 Results

### 3.1 Phenotypic characteristics

**Figure 1:**
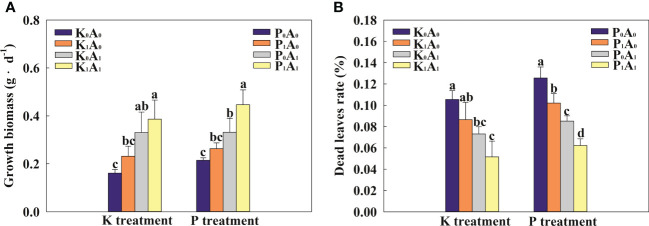
Effects of *Aspergillus aculeatus* on growth biomass **(A)** and death leaf rate **(B)** of bermudagrass under K or P deficiency stress. K_0_A_0_ represents no potassium feldspar and *A. aculeatus* treatment; K_1_A_0_ represents only potassium feldspar treatment; K_0_A_1_ represents only *A. aculeatus* treatment; K_1_A_1_ represents potassium feldspar + *A. aculeatus* treatment; P_0_A_0_ represents no tricalcium phosphate and *A. aculeatus* treatment; P_1_A_0_ represents only tricalcium phosphate treatment; P_0_A_1_ represents only *A. aculeatus* treatment; P_1_A_1_ represents tricalcium phosphate + *A. aculeatus* treatment. Columns marked with the same small letter indicate insignificant differences between the four treatment groups (*p* < 0.05).

Phosphorus and potassium play an important role in the growth and development of plants. As shown in **Figure 1A**, the growth biomass of bermudagrass was significantly increased by 105.4% in the K_0_A_1_ group compared with the K_0_A_0_ treatment. In addition, the K_1_A_1_ treatment significantly enhanced the growth of bermudagrass by 66.7% compared with the K_1_A_0_ treatment. At the same time, bermudagrass biomass had an obvious increase (by 69.7%) in the P_1_A_1_ group compared with the P_1_A_0_ regime. In the K treatment group, compared with the K_0_A_0_ group, the dead leaf rate of bermudagrass was significantly reduced by 44.3% in the K_0_A_1_ group (**Figure 1B**). K_1_A_1_ treatment significantly improved the growth of bermudagrass under the K_1_A_0_ stress, which showed a significant decrease of 40.4% in the dead leaf rate. Similarly, P_1_A_1_ treatment showed a significant decrease of 39.0% in the rate of dead leaf compared with the P_1_A_0_ group. Taken together, *A. aculeatus* could enhance the growth situation of bermudagrass by improving biomass and decreasing the dead leaf rate under P- or K-deficient conditions.

### 3.2 Chlorophyll and carotenoid contents of bermudagrass

**Figure 2:**
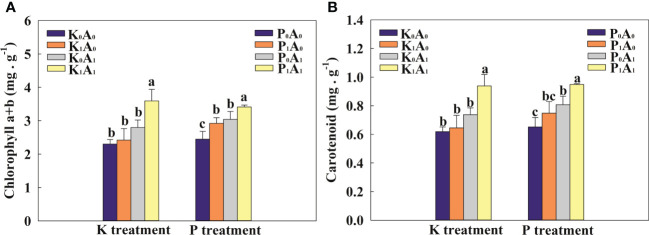
Effects of *Aspergillus aculeatus* on chlorophyll **(A)** and carotenoid **(B)** contents of bermudagrass under K or P deficiency stress. K_0_A_0_ represents no potassium feldspar and *A. aculeatus* treatment; K_1_A_0_ represents only potassium feldspar treatment; K_0_A_1_ represents only *A. aculeatus* treatment; K_1_A_1_ represents potassium feldspar + *A. aculeatus* treatment; P_0_A_0_ represents no tricalcium phosphate and *A. aculeatus* treatment; P_1_A_0_ represents only tricalcium phosphate treatment; P_0_A_1_ represents only *A. aculeatus* treatment; P_1_A_1_ represents tricalcium phosphate + *A. aculeatus* treatment. Columns marked with the same small letter indicate insignificant differences between the four treatment groups (*p* < 0.05).

Under K-deficient conditions, the chlorophyll a+b and carotenoid contents were not obviously different in the K_0_A_0_, K_1_A_0_, and K_0_A_1_ groups (**Figure 2**). Nevertheless, the K_1_A_1_ treatment significantly increased the chlorophyll a+b and carotenoid contents of bermudagrass by 48.5% and 45.4%, respectively, compared with the K_1_A_0_ treatment. Similarly, under P-deficient conditions, the chlorophyll a+b and carotenoid contents were increased by 30.1% and 23.6% in inoculated plants (P_0_A_1_ treatment) compared with the P_0_A_0_ treatment. Compared with the P_1_A_0_ treatment, the chlorophyll a+b and carotenoid contents in the P_1_A_1_ group increased remarkably (16.7% and 26.9%, respectively) (**Figure 2**). These results implied that *A. aculeatus* promoted the biosynthesis of chlorophyll a+b and carotenoid contents through its P- or K-releasing characteristics.

### 3.3 Forage quality

**Figure 3:**
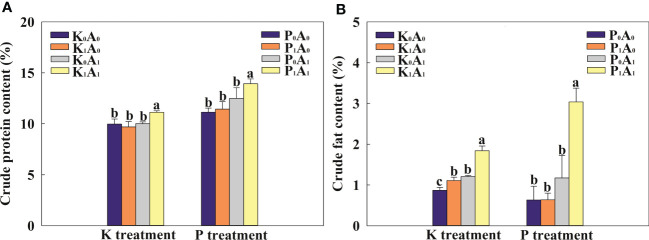
Effects of *Aspergillus aculeatus* on crude protein **(A)** and crude fat **(B)** contents of bermudagrass under K or P deficiency stress. K_0_A_0_ represents no potassium feldspar and *A. aculeatus* treatment; K_1_A_0_ represents only potassium feldspar treatment; K_0_A_1_ represents only *A. aculeatus* treatment; K_1_A_1_ represents potassium feldspar + *A. aculeatus* treatment; P_0_A_0_ represents no tricalcium phosphate and *A. aculeatus* treatment; P_1_A_0_ represents only tricalcium phosphate treatment; P_0_A_1_ represents only *A. aculeatus* treatment; P_1_A_1_ represents tricalcium phosphate + *A. aculeatus* treatment. Columns marked with the same small letter indicate insignificant differences between the four treatment groups (*p* < 0.05).

The *A. aculeatus* treatment obviously increased the crude fat content of bermudagrass by 38.6% compared with the K_0_A_0_ treatment and had no effect on crude protein content under K deficiency stress (**Figure 3**). However, *A. aculeatus* inoculations combined with K-feldspar (K_1_A_1_ regime) remarkably elevated the crude protein and crude fat content by 14.9% and 65.7%, respectively, in bermudagrass, compared with the K-feldspar treatment (K_1_A_0_ group). Similarly, under P-deficient conditions, the P_1_A_1_ treatment significantly increased the crude protein and crude fat contents by 21.8% and 377.7%, respectively, compared with the P_1_A_0_ treatment. In the environment of K or P deficiency, exogenous *A. aculeatus* effectively increased the contents of crude protein and crude fat and then improved the forage quality of bermudagrass.

### 3.4 Ion homeostasis

**Figure 4:**
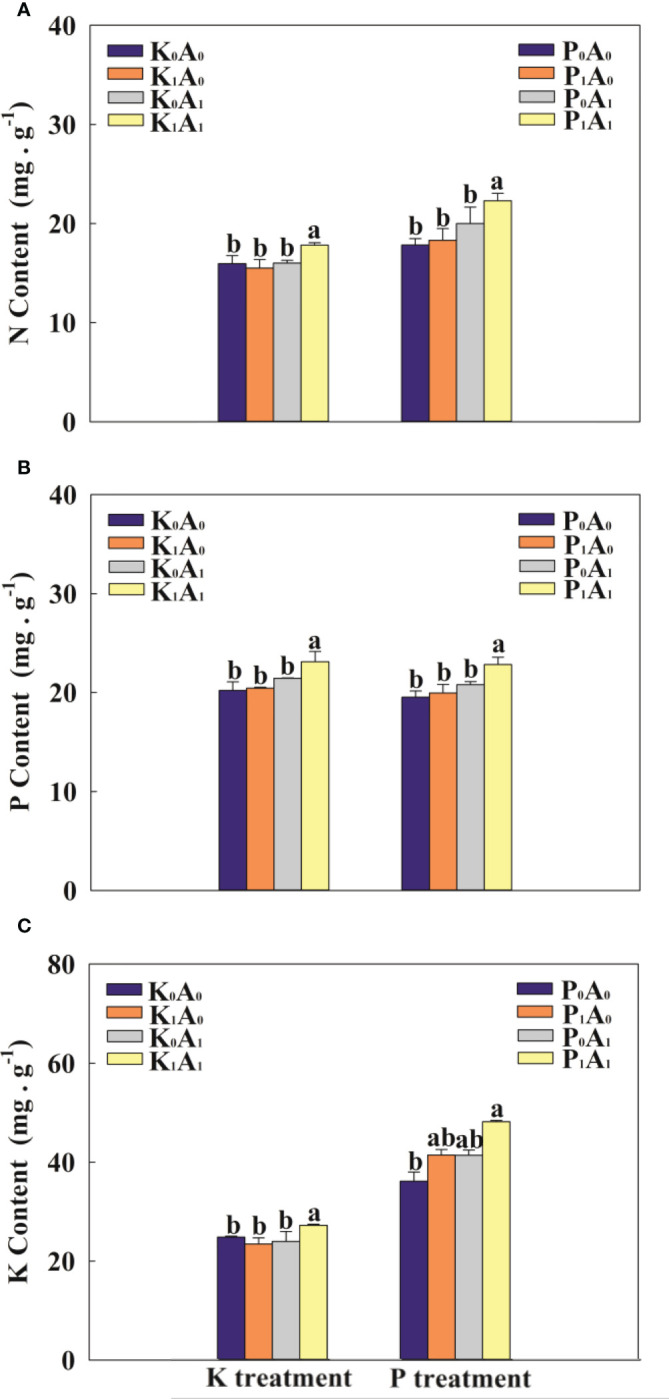
Effects of *Aspergillus aculeatus* on N content **(A)**, P content **(B)** and K content **(C)** of bermudagrass under K or P deficiency stress. K_0_A_0_ represents no potassium feldspar and *A. aculeatus* treatment; K_1_A_0_ represents only potassium feldspar treatment; K_0_A_1_ represents only *A. aculeatus* treatment; K_1_A_1_ represents potassium feldspar + *A. aculeatus* treatment; P_0_A_0_ represents no tricalcium phosphate and *A. aculeatus* treatment; P_1_A_0_ represents only tricalcium phosphate treatment; P_0_A_1_ represents only *A. aculeatus* treatment; P_1_A_1_ represents tricalcium phosphate + *A. aculeatus* treatment. Columns marked with the same small letter indicate insignificant differences between the four treatment groups (*p* < 0.05).

Compared with K_0_A_0_, the K_1_A_0_ and K_0_A_1_ treatments had no obvious effect on the contents of N, P, and K in the leaves (**Figure 4**). The K_1_A_1_ treatment markedly enhanced the contents of N, P, and K in the leaves of bermudagrass by 14.9%, 13.2%, and 16.1%, respectively, compared with the K_1_A_0_ treatment. Under P-deficient conditions, the P_1_A_1_ treatment significantly increased the contents of N, P, and K in the leaves of bermudagrass by 21.8%, 14.4%, and 16.3%, respectively, compared with the P_1_A_0_ group (**Figure 4**). These results suggest that *A. aculeatus* can dissolve insoluble K and P into soluble K and P, thereby promoting the uptake of K and P and enhancing the contents of N, P, and K in the leaves of bermudagrass.

### 3.5 Antioxidant system

**Figure 5:**
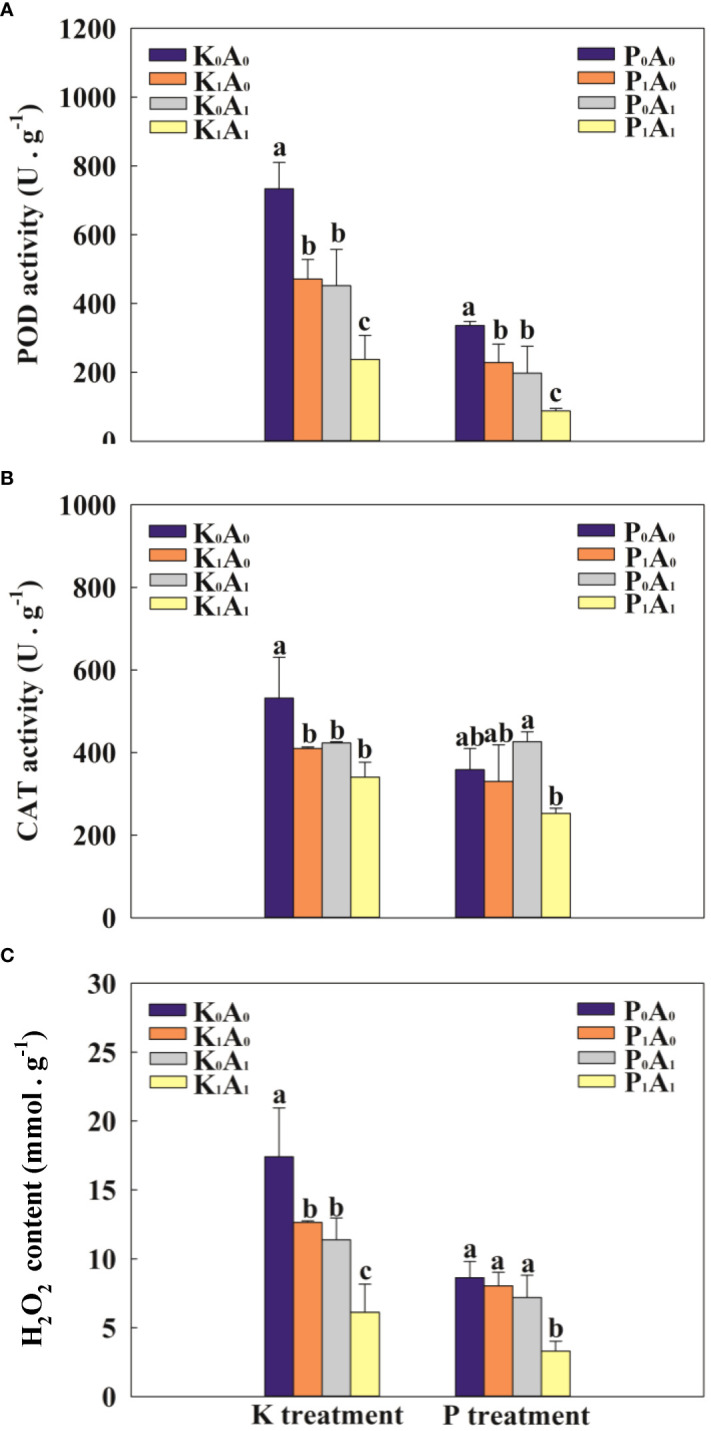
Effects of *Aspergillus aculeatus* on POD activity **(A)**, CAT activity **(B)** and H_2_O_2_ content **(C)** of bermudagrass under K or P deficiency stress. K_0_A_0_ represents no potassium feldspar and *A. aculeatus* treatment; K_1_A_0_ represents only potassium feldspar treatment; K_0_A_1_ represents only *A. aculeatus* treatment; K_1_A_1_ represents potassium feldspar + *A. aculeatus* treatment; P_0_A_0_ represents no tricalcium phosphate and *A. aculeatus* treatment; P_1_A_0_ represents only tricalcium phosphate treatment; P_0_A_1_ represents only *A. aculeatus* treatment; P_1_A_1_ represents tricalcium phosphate + *A. aculeatus* treatment. Columns marked with the same small letter indicate insignificant differences between the four treatment groups (*P* < 0.05).

Adverse environments can trigger the excessive accumulation of ROS and aggravate lipid peroxidation in plants. Compared with K_0_A_0_, the POD, CAT, and H_2_O_2_ of bermudagrass in the K_1_A_0_ treatment group were significantly reduced by 55.8%, 30.4%, and 37.8%, while in the K_0_A_1_ treatment group, the POD, CAT, and H_2_O_2_ activities of bermudagrass were significantly reduced by 62.6%, 25.8%, and 184.2%, respectively (**Figure 5**). Compared with the K_1_A_0_ treatment, the POD activity and H_2_O_2_ level were significantly decreased by 49.5% and 51.5%, respectively, in the K_1_A_1_ treatment. Simultaneously, under P deficiency stress, the P_1_A_1_ treatment significantly decreased the POD activity and H_2_O_2_ content (by 61.6% and 59.0%, respectively), compared with the P_1_A_0_ treatment. These results indicate that under the P- or K-deficient conditions, *A. aculeatus* might decrease the activity of the antioxidant enzymes CAT and POD and alleviate membrane lipid peroxidation.

### 3.6 Correlation analysis

**Figure 6:**
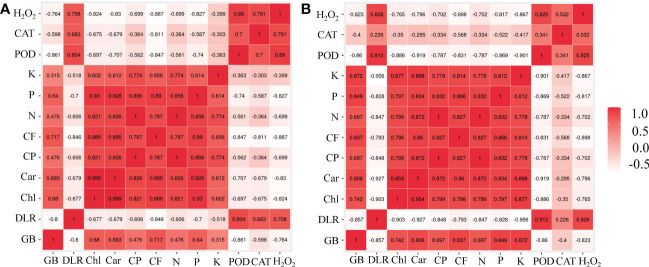
Correlation plot designed by the R software for all measured traits in bermudagrass under K deficiency **(A)** and P deficiency stress **(B)**. In the plots, red, pink, and white represent positive, zero, and negative correlations, respectively, and the darker the color (either red or white), the stronger the correlation. GB, growth biomass; DLR, dead leaf rate; Chl, chlorophyll a+b; Car, carotenoid; CP, crude protein; CF, crude fat; N, N content; P, P content; K, K content; POD, peroxidase; CAT, catalase; H_2_O_2_, hydrogen peroxide.

Two correlation plots were depicted for each K or P deficiency stress treatment and are presented in **Figures 6A, B**, respectively. These correlation plots provide an observation to visually compare the correlation of measured traits. Under K or P deficiency stress, the growth biomass was significantly positively correlated with chlorophyll a+b, carotenoid, and P contents and negatively correlated with the dead leaf rate (**Figure 6**). There was a significant positive correlation between forage quality (crude protein and crude fat) and chlorophyll a+b, carotenoids, P content, K content, and N content in the leaves of bermudagrass. However, the phenotypic indicators (growth biomass, chlorophyll a+b, carotenoid, crude protein, crude fat, N content, P content, and K content) showed a significant negative correlation with H_2_O_2_ content.

## 4 Discussion

Nitrogen, phosphorus, and potassium play a fundamental role in the growth and development of plants, and they are important mineral nutrients involved in photosynthesis (Wang et al., 2012; Ding et al., 2021). Under the conditions of P or K deficiency, the accumulation of N, P, and K in plants decreases, thereby inhibiting the absorption and utilization of nutrients and impeding the photosynthesis of plants. Photosynthetic pigments are one of the key factors affecting photosynthesis, and their content can directly affect the photosynthesis of plants, particularly chlorophyll and carotenoid contents (Bode et al., 2009). Our results showed that under P or K deficiency stress, the contents of N, P, and K in the leaves increased significantly when *A*. *aculeatus* was inoculated. Previous studies have confirmed the P- and K-solubilizing activities of *A*. *aculeatus* (Li et al., 2019; Li et al., 2021). In addition, the chemical characteristics of soils with poor fertility have also been measured. We found that the content of available P, K, total N, and total K in the soil was significantly increased after inoculating *A. aculeatus* into the soil with poor fertility (unpublished data). The beneficial effect of *A*. *aculeatus* amendment on bermudagrass under P or K deficiency supports earlier studies. The research reported that co-inoculation of P- and K-solubilizing microorganisms in conjunction with the direct application of insoluble P and K into the soil enhanced N, P, and K uptake of plants grown on P- and K-limited soils (Meena et al., 2016). As we observed, the contents of chlorophyll a+b and carotenoids were distinctly increased in *A*. *aculeatus*-inoculated bermudagrass (P_1_A_1_ and K_1_A_1_ groups) compared with the other groups and were significantly positively correlated with N, P, and K, as well as significantly negatively correlated with the dead leaf rates. The correlation between these functional traits could provide a relatively comprehensive evaluation of plant adaptability to P or K deficiency stress. This is fully aligned with a previous study, which documented that inoculation with a P-solubilizing strain stimulates an increase in chlorophyll (chl a and b) content and nutrient uptake (N, P, and K) in plants (Marathe et al., 2017). Overall, we suggested that *A*. *aculeatus* could contribute to better photosynthetic activity (increased chlorophyll a and b content) and growth characteristics when plants are exposed to P or K deficiency stress.

Forage quality is one of the indicators for evaluating the value of forage utilization. A higher feed value was accompanied by a higher crude protein and crude fat content in pasture (Li et al., 2018). Studies have demonstrated that N, P, and K effectively enhanced the content of crude protein and crude fat in plants (Osuagwu and Edeoga, 2013; Ogunyemi et al., 2018). Compared with P- or K-deficient treatments, the contents of crude protein and crude fat were markedly enhanced in the leaves of bermudagrass inoculated with *A*. *aculeatus* and were significantly positively correlated with the N and P contents. Nitrogen is an important component of chlorophyll and protein in plants, and a sufficient nitrogen supply can increase the content of chlorophyll and soluble protein (Amy et al., 2006; Fredeen et al., 2010), which is consistent with our results. In addition, P is a component of a series of important biochemical substances, such as nucleic acids, coenzymes, phosphoproteins, and phospholipids, and its deficiency affects nitrogen metabolism. According to Baier and Kristan (1986), the application of P could increase the uptake of N by plants, thereby enhancing the content of crude protein and forage quality (Baier and Kristan, 1986). In the same way, K deficiency could reduce the energy conversion rate and, thus, decrease the crude protein content of herbage, leading to the degradation of forage quality. Combining the above results, we concluded that the inoculation of *A*. *aculeatus* enhanced plant forage quality by improving P and K solubilizers. Enhancement of plant forage quality by improving P and K solubilizers is another beneficial effect of microorganisms with P- and K-solubilizing potential characteristics.

In this study, important indicators such as POD, CAT, and H_2_O_2_ were assessed to investigate the role of *A. aculeatus* for P or K deficiency stress tolerance in bermudagrass. When plants are exposed to abiotic stresses, increased cellular damage is associated with the production of ROS (Fadzilla et al., 1997). In our results, we found that inoculated bermudagrass showed remarkably lower H_2_O_2_ levels than uninoculated bermudagrass under P- or K-deficient conditions, indicating that *A. aculeatus* could significantly attenuate stress-induced oxidative damage. Although plants cannot escape from deleterious environments, they have already developed and established a mathematical regulatory mechanism to cope with stress damage (Abeed et al., 2022). Usually, stress-induced ROS accumulation is scavenged by diverse enzymatic scavengers, such as POD, CAT, SOD, or other antioxidants (Apel and Hirt, 2004). H_2_O_2_ is an important ROS produced in the catalytic process of SOD, resulting in oxidative damage (Mittler, 2002). CAT and POD are the major antioxidant enzymes in plants, and CAT could degrade H_2_O_2_ into water and oxygen to scavenge H_2_O_2_. Our results indicated that inoculation with *A. aculeatus* significantly decreased the CAT and POD activities and the concentration of H_2_O_2_ in the P_1_A_1_ and K_1_A_1_ groups compared with the P_1_A_0_ and K_1_A_0_ groups. These studies reveal that *A. aculeatus* can alleviate the antioxidant damage caused by low phosphorus or potassium stress by decreasing ROS accumulation and altering the activity of antioxidant enzymes.

Therefore, our results demonstrated the regulatory mechanism of *A. aculeatus* on K or P deficiency stresses in bermudagrass. First, *A. aculeatus* increases the contents of N, P, and K in the leaves of bermudagrass and enhances the accumulation of chlorophyll and carotenoids, thereby promoting plant photosynthesis. Second, *A. aculeatus* increases the content of crude protein and crude fat accompanied by the growing N and P contents in the leaves of bermudagrass. Finally, *A. aculeatus* could mitigate membrane lipid peroxidation and decrease the activities of the antioxidant enzymes CAT and POD. Therefore, *A. aculeatus* has great promotion value and economic benefit in K- or P-deficient soil as a microbial agent.

## Data availability statement

The original contributions presented in the study are included in the article/supplementary material. Further inquiries can be directed to the corresponding author.

## Author contributions

XL conceived the experiments and wrote the manuscript. TZ performed the experiments. XL and XX analyzed the data. YX and XC cultivated the experimental materials. JF guided this experiment. All authors contributed to the article and approved the submitted version.
